# Xanthogranulomatous pyelonephritis mimicking renal cell carcinoma: a case report

**DOI:** 10.1097/MS9.0000000000000474

**Published:** 2023-04-03

**Authors:** Nagham Daoud, Nahar Ismaiel, George Bashour, Ali Nammour, Ali Barri, Zuheir Alshehabi

**Affiliations:** a Cancer Research Center; b Faculty of Medicine; c Department of Urology; d Department of Pathology, Tishreen University Hospital, Latakia, Syria

**Keywords:** case report, diagnosis, immunohistochemistry, renal cell carcinoma, xanthogranulomatous pyelonephritis

## Abstract

Xanthogranulomatous pyelonephritis (XGPN) is a rare, serious, and chronic inflammatory disorder of the kidney characterized by a destructive process that invades the renal parenchyma, which is most commonly associated with urinary tract obstruction and infection. It affects women more often than men.

**Case presentation:**

Herein, the authors report a case of a 48-year-old male presented to their hospital with complaints of malaise, fever, chills, left flank pain, and a history of a staghorn calculus in the renal pelvis, which was removed by surgery 7 years ago. Ultrasonography and computed tomography scans showed an enlarged left kidney with cystic formation and pelvicalyceal system dilation with the presence of multiple large stones. The renogram showed a dysfunctioning left kidney. An open radical left nephrectomy was performed. Renal cell carcinoma (RCC) was suspected in both the gross and microscopic examinations. The immunohistochemistry was the decisive factor in confirming the diagnosis of XGPN.

**Clinical discussion:**

Preoperative and postoperative diagnosis of XGPN can sometimes be difficult due to diverse differential diagnoses. The misinterpretation of ‘foam cells’ as ‘clear cells’ consistent with RCC is the most important diagnostic challenge for pathologists.

**Conclusion:**

The unusual findings of this case report suggest a careful evaluation of patients with a renal cystic mass, that can be misdiagnosed as a RCC. A combined computed tomography scan evaluation together with histopathology and immunohistochemistry are essential for a correct diagnosis of this rare renal entity.

## Introduction

HIGHLIGHTS

- Xanthogranulomatous pyelonephritis (XGPN) is a rare, serious, and chronic inflammatory disorder of the kidney characterized by a destructive mass that invades the renal parenchyma.
- Histologically, the main differential diagnosis is clear cell renal cell carcinoma, whose cells have clear cytoplasm that may resemble histiocytes.
- The main learning point of our case is that pathological examination using routine stains (H&E) does not always provide a definitive diagnosis of XGPN. The histopathological features of clear cell renal cell carcinoma and XGPN may pose a diagnostic dilemma. This is mainly because foamy histiocytes may be confused with neoplastic clear cells.
- The exclusion of epithelial markers by immunohistochemistry should resolve any uncertainty.
- A differential diagnosis of XGPN must be considered to prevent inadequate and unnecessary treatment.

Xanthogranulomatous pyelonephritis is a rare, chronic inflammatory destructive process of renal parenchyma^1^.

Most cases of XGPN are often associated with urinary tract obstruction, infection, and/or nephrolithiasis. XGPN is four times more common in women than men and is usually detected in the fifth and sixth decades of life. XGPN affects both kidneys with equal frequency^2^.

Some studies have classified XGPN as focal, segmental, and diffuse^1^ while others have classified it as being either diffuse or focal with the focal form imitating greatly renal cell carcinoma (RCC)^3^.

We present a rare challenging case of XGPN, which was initially interpreted as a RCC based on the first gross and microscopic impression. However, immune stains were essential to rule out malignancy and confirm the inflammatory nature of the lesion.

This case report has been reported in line with the Surgical CAse REport (SCARE) criteria^4^.

## Case presentation

A 48-year-old male presented to the emergency department at our hospital with malaise, fever, chills, and left flank pain with no reported drug or food-related allergies.

On examination, a large palpable mass was located in the left flank with tenderness in that region. The physical exam showed no other remarkable findings.

Seven years ago, the patient was diagnosed with a staghorn calculus in the left renal pelvis. Surgery was performed to remove the calculus. This was followed by several rounds of lithotripsy. Nevertheless, the patient neglected his condition and did not complete his treatment.

Laboratory findings showed an elevated white blood count (WBC) count (16 850/ml) with high neutrophils (82.2%) and a high C Reactive Protein (CRP) (197 mg/dl). Ultrasonography showed an enlarged left kidney (longitudinal diameter of 20 cm) with a thin renal cortex (2 mm) and severe chronic dilation of the collecting system and the renal pelvis. The upper ureter was dilated (18 mm) and it contained a calculus (18 mm) that was located 8 cm below the renal pelvis.

The preliminary diagnosis was pyelonephritis. The patient was admitted to the urology department for further investigation and was treated with antibiotics.

A dynamic scan of the kidneys using the radioactive tracer 99 mTc-MAG3 was done, and it showed asymmetrical flow to both kidneys; decreased on the left renal angiogram. The renogram of the left kidney demonstrated heterogeneous faint cortical uptake with a delay in radiotracer excretion. The left kidney showed faint radiotracer uptake by the end of the study and the left collecting system was not visualized. The renogram of the right kidney showed homogenous cortical uptake and normal corticomedullary transit. The right kidney was within normal range.

The split renal function was 92% for the right kidney and 8% for the left kidney.

**Figure 1:**
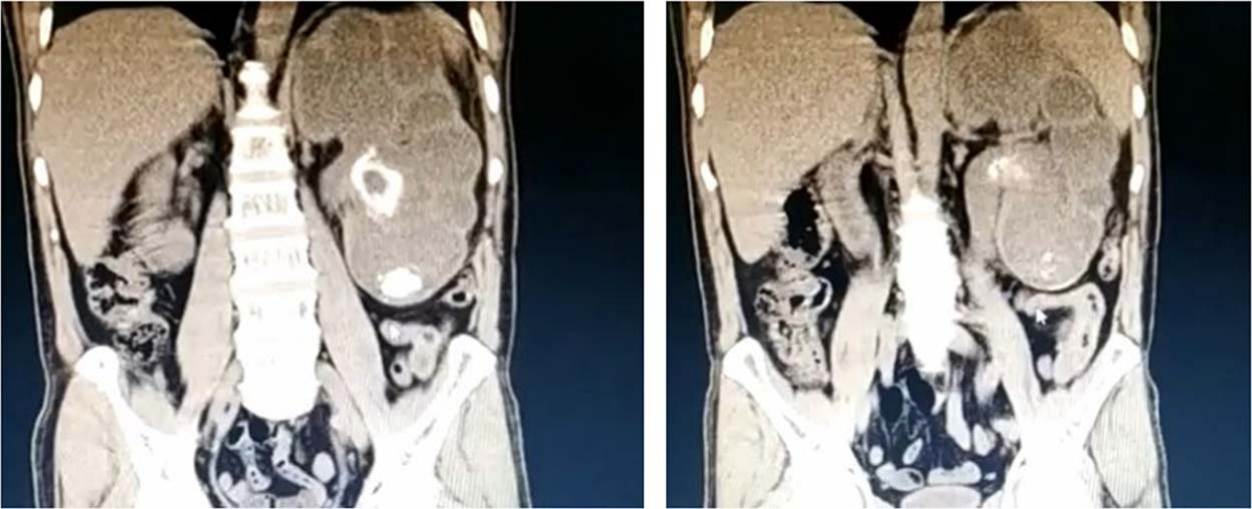
A noncontrast computed tomography sagittal view of the abdomen showing the calculus in the renal pelvis and other obstructing calculi.

**Figure 2:**
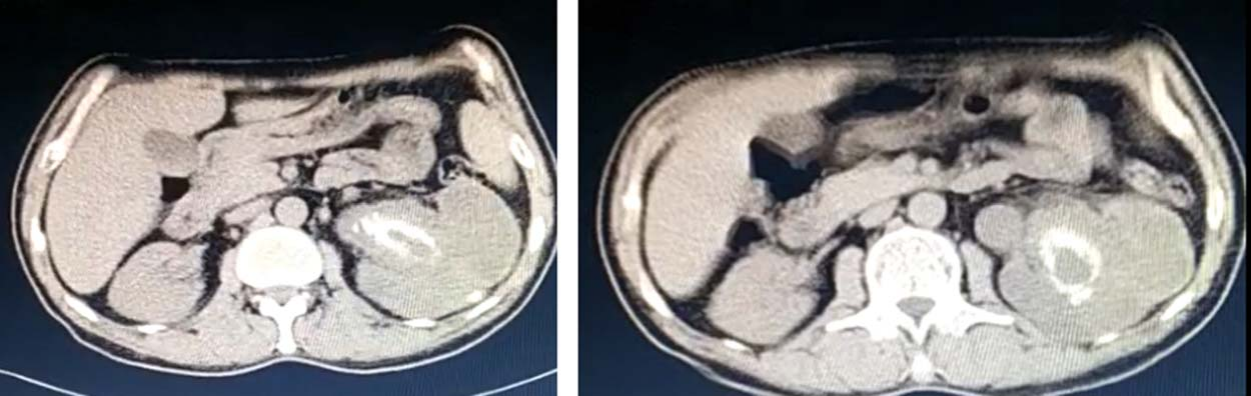
A noncontrast computed tomography axial view of the abdomen showing the enlarged left kidney with cystic pelvicalyceal dilation.

A computed tomography (CT) scan of the abdomen and pelvis without contrast (Figs. 1 and 2) was performed and it showed an enlarged left kidney with cystic formation measuring 4×5 cm in maximum dimensions and dilation of the pelvicalyceal system with the presence of multiple variable-sized calculi. In addition, there was a large stone seen impacted at the proximal third of the ureter measuring 1.6 cm. The right kidney was normal in size and position.

24 hours later, after the antibiotic treatment, the decision was made for the patient to undergo an open radical left nephrectomy to remove the dysfunctioning left kidney and to drain the abscess.

The surgery was performed by a senior surgeon with a left subcostal incision, and the surgeon encountered dense adhesions due to the prior operation. The renal artery and accompanying vein were tied and the kidney was resected.

The surgical site was flushed with saline and a silicone drainage tube (18 Fr) was placed until postop day four. The patient felt well immediately after the operation and all the related symptoms subsided.

The patient had a postoperative wound infection with a raised WBC count 34 800/ml and a CRP 150 mg/dl. Due to that, the patient was put on broad-spectrum antibiotics (metronidazole, ceftriaxone, and levofloxacin) and monitored closely until WBC count and CRP went back to normal.

A macroscopic exam showed an enlarged left kidney measuring (17×11×8) cm with a thickened capsule and yellow nodules. The resected kidney was opened and it showed cystic formations; some of which contained necrotic debris and others contained thrombi, especially in the lower pole. Pathological examination of the cystic yellow areas on routine stain (H&E) showed sheets of large to medium-sized cells with clear cytoplasm and central nuclei with scattered inflammatory cell exudates. The differential diagnosis based on gross and microscopic impression was clear cell RCC.

**Figure 3:**
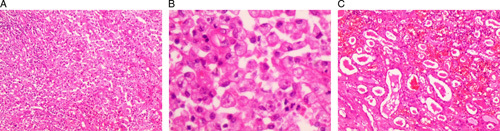
(A) XGPN, diffuse proliferation of foamy histiocytes replacing renal parenchyma with mixed inflammatory exudates. H&E×100. (B) XGPN, Foamy histiocytes with central nuclei and abundant clear cytoplasm. H&E×400. (C) XGPN, tubular thyroidisation (filled with colloid casts) and tubular atrophy. H&E×100.

**Figure 4:**
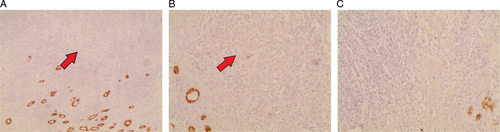
(A) Immunohistochemistry with cytokeratin (the arrow shows negativity for computed tomography in the affected area of the kidney) whereas the residual renal tissue is positive for computed tomography. (B) Immunohistochemistry with cytokeratin-7 (the arrow shows negativity for cytokeratin-7 in the affected area of the kidney). (C) Immunohistochemistry showing negativity for cytokeratin-20 in the affected area of the kidney.

To confirm the diagnosis; immune stains were recommended (Figs. 3 and 4). The suggested panel includes (CK, CK7, and CK20) and the suspected ‘clear cells’ were negative for CK, CK7, and CK20, which ruled out the diagnosis of RCC. The ‘foam cells’ consistent with XGPN were first misinterpreted as malignant ‘clear cells’. The immunohistochemistry study was essential to rule out malignancy and confirmed the diagnosis of XGPN. CD68 was not available in our pathology lab.

## Discussion

XGPN is a serious chronic inflammatory kidney disease that leads to focal or diffuse renal destruction. If uncontrolled, it spreads to adjacent tissue (perinephric fat and retroperitoneal space) and destroys it^1^,^5^.

Most cases of XGPN are often associated with urinary tract obstruction, infection, and/or nephrolithiasis, and common symptoms are flank or abdominal pain, fever, palpable mass, lower urinary tract symptoms, total hematuria, and weight loss^2^.

The most typical CT findings of XGPN are unilateral renal enlargement and extra renal extension of inflammatory changes. The presence of these features in a patient with common symptoms and urinary tract obstruction, and infection should orientate the radiologist towards the possibility of XGPN^6^.

XGPN has been described as a great imitator or a masquerading tumor in adults and pediatric age groups^7^. After the microscopic examination, XGPN can be misdiagnosed with a renal cell tumor.

The macroscopic appearance of the resected kidney in XGPN usually shows an enlarged kidney with a thickened capsule, yellow masses with variable central necrosis, and a dilated renal pelvis with stones or purulent material. The mass lesions of XGPN may grossly elicit concern for a neoplasm.

The microscopic examination of XGPN usually shows replacement of renal parenchyma with CD68+ foamy histiocytes, occasional multinucleated giant cells and inflammatory cells.

Histologically, the main differential diagnosis is clear cell RCC, whose cells have clear cytoplasm that may resemble histiocytes, but are usually in compact, tubulocystic or alveolar patterns accompanied by a delicate, chicken wire vasculature. In addition, the bubbly microvesicular fat of the foamy histiocytes contrasts with the cleared-out cytoplasm that is characteristic of clear cell RCC. The RCC cells often contain glassy hyaline globules, have nuclear grades 2–3, are cytokeratin-positive and CD68-negative.

The exclusion of epithelial markers by immunohistochemistry should resolve any uncertainty. The xanthoma cells are CD68-positive and cytokeratin-negative, and the reactive fibrous tissue is vimentin positive and cytokeratin-negative^1^.

Our case was a pathological challenge. Both gross and pathological examination (H&E stain) suggested RCC; therefore, this stresses the importance of immunohistochemistry using cytokeratin, which was ordered (CD68 was not available in our lab) to provide a definitive diagnosis and to rule out RCC.

Several deaths have been reported directly from XGPN due to postoperative sepsis^8^. Therefore, postoperative broad-spectrum antibiotics are highly recommended^1^. Our case has stressed the importance of this procedure as the patient was given broad-spectrum antibiotics.

The main learning point of our case is that pathological examination using routine stains (H&E) does not always provide a definitive diagnosis of XGPN. The histopathological features of clear cell RCC and XGPN may pose a diagnostic dilemma. This is mainly because foamy histiocytes may be confused with neoplastic clear cells. XGPN has also been found to be mistaken with other types of malignancy, including papillary transitional cell carcinoma and squamous cell carcinoma; hence, this case assures that the final diagnosis should be determined by immunohistochemistry studies especially when malignancy cannot be excluded.

Immune stains can reduce the probability of a false diagnosis of RCC in XGPN cases and distinguish the entities.

Finally, a differential diagnosis of XGPN must be considered to prevent inadequate and unnecessary treatment. Also, in the differential diagnosis of renal masses developed in the background of chronic obstruction, cases of XGPN should be kept in mind. A high level of awareness is needed to accomplish the right diagnosis.

## Conclusion

We present a rare case report of Xanthogranulomatous Pyelonephritis, which was initially interpreted as a RCC based on the first gross and microscopic impression. Microscopically, misinterpretation of ‘foam cells’ as ‘clear cells’ consistent with RCC is the most important diagnostic challenge for pathologists.

The unusual findings of this case report suggest a careful evaluation of patients with a renal cystic mass that can be misdiagnosed as a RCC. A combined CT-scan evaluation together with histopathology and immunohistochemistry are essential for a correct diagnosis of this rare renal entity.

## Ethical approval

Given the nature of the article, a case report, no ethical approval was required.

## Consent

Written informed consent was obtained from the patient for publication of this case report and accompanying images. A copy of the written consent is available for review by the Editor-in-Chief of this journal on request.

## Sources of funding

No funding was required.

## Author contributions

N.D., N.I., and G.B.: writing - original draft, reviewing, and editing; A.N. and A.B.: supervision, reviewing, and editing; Z.A.: supervision, final reviewing, and editing. All authors contributed to this manuscript.

## Conflict of interest disclosure

The authors declare no conflict of interest.

## Research registration unique identifying number (UIN)

Not needed.

## Guarantor

Professor Zuheir Alshehabi.

## Provenance and peer review

Not commissioned, externally peer-reviewed.
